# Beyond Chronological Age: Frailty, Vulnerability, and Invasive Decision-Making in Older Adults with Acute Coronary Syndromes

**DOI:** 10.3390/geriatrics11040106

**Published:** 2026-08-17

**Authors:** Lourdes Vicent, Rafael Salguero-Bodes, Pablo R. Alonso, Helena Alarcos, Elena Puerto García-Martín, Carlos Diaz-Arocutipa, Fernando Arribas Ynsaurriaga, Roberto Martín-Asenjo

**Affiliations:** 1 Department of Cardiology, Hospital Universitario 12 de Octubre, 28041 Madrid, Spain; 2 Centro de Investigación Biomédica en Red Enfermedades Cardiovasculares (CIBERCV), 28029 Madrid, Spain; 3 Facultad de Medicina, Universidad Complutense, 28041 Madrid, Spain; 4 Comité de Ética de la Investigación con Medicamentos, Hospital 12 de Octubre, 28041 Madrid, Spain; 5 Unidad de Revisiones Sistemáticas y Meta-Análisis (URSIGET), Vicerrectorado de Investigación, Universidad San Ignacio de Loyola, Lima 15024, Peru

**Keywords:** acute coronary syndromes, frailty, older adults, invasive management, vulnerability, geriatric cardiology, therapeutic intensity, patient-centered outcomes, healthcare inequities, shared decision-making

## Abstract

Older adults represent a growing proportion of patients presenting with acute coronary syndromes (ACS), yet they remain a highly heterogeneous population in terms of biological reserve, comorbidity burden, functional status, cognitive performance, and recovery potential. Chronological age alone is an insufficient basis for invasive decision-making, as it may lead to both therapeutic nihilism and disproportionate treatment escalation. Frailty has emerged as a clinically meaningful construct that captures vulnerability to acute stressors and may refine prognostic assessment beyond traditional cardiovascular risk scores. In ACS, frailty is associated with mortality, bleeding, procedural complications, delirium, functional decline, readmission, and loss of independence. However, frailty should not be interpreted as an automatic contraindication to invasive management. Rather, it should inform proportional care by integrating ischemic risk, procedural burden, reversibility potential, patient preferences, and expected quality of recovery. This narrative review examines the role of frailty assessment in older adults with ACS, focusing on its implications for invasive decision-making. We discuss frailty tools, clinical outcomes, therapeutic bias, healthcare inequities, and patient-centered endpoints. Finally, we propose a vulnerability-based framework for cardiovascular care, in which frailty guides individualized therapeutic intensity rather than justifying age-based exclusion from evidence-based treatment.

## 1. Introduction

Acute coronary syndromes (ACS) are increasingly encountered in older adults, reflecting population aging, improved survival with chronic cardiovascular disease, and the accumulation of comorbidity across the life course. Older patients represent one of the highest-risk groups within contemporary ACS care, with greater rates of mortality, bleeding, renal dysfunction, delirium, functional decline, readmission, and institutionalization after hospitalization [1,2,3]. At the same time, they are markedly heterogeneous. Two patients of the same chronological age may differ profoundly in biological reserve, cognitive status, mobility, comorbidity burden, social support, treatment tolerance, and likelihood of meaningful recovery. This heterogeneity makes invasive decision-making particularly complex.

Current ACS pathways are largely structured around ischemic risk, hemodynamic status, electrocardiographic findings, biomarkers, coronary anatomy, and procedural feasibility [4]. These dimensions remain essential. However, in older adults, they may be insufficient to determine the most appropriate therapeutic strategy. Chronological age is frequently used, explicitly or implicitly, as a surrogate for vulnerability, procedural risk, or limited benefit. This may lead to undertreatment when older patients are denied coronary angiography or revascularization solely because of age, but also to overtreatment when invasive strategies are pursued without adequate consideration of frailty, functional trajectory, patient goals, or competing non-cardiovascular risks. Importantly, the integration of frailty assessment should not delay evidence-based reperfusion or invasive management in time-critical presentations such as ST-segment elevation myocardial infarction. Rather, frailty assessment should complement guideline-directed ACS care by informing individualized management after initial stabilization and throughout the subsequent phases of treatment and recovery.

This tension reflects a broader therapeutic paradox in geriatric cardiology. Older adults often have the highest absolute ischemic risk and may therefore have the greatest potential to benefit from effective treatment. Yet they are also more likely to experience harm from invasive procedures, antithrombotic therapy, prolonged hospitalization, and treatment-related complications [5]. Moreover, older and frail patients have historically been underrepresented in randomized clinical trials, limiting the external validity of evidence that informs guideline recommendations [6]. As a result, clinicians frequently face decisions in which guideline-directed care, procedural risk, patient values, and expected recovery do not align neatly.

Frailty has emerged as a key concept to address this gap. Frailty is commonly understood as a state of reduced physiological reserve and increased vulnerability to stressors, resulting from the cumulative decline of multiple physiological systems [7]. In the context of ACS, frailty may influence not only prognosis but also the balance between ischemic benefit and procedural burden. It may identify patients at increased risk of bleeding, acute kidney injury, delirium, prolonged hospitalization, functional deterioration, and poor quality of recovery [8,9,10]. Importantly, frailty captures a dimension of risk that is not fully reflected by traditional cardiovascular scores.

However, the clinical use of frailty assessment requires caution. Frailty should not become a shorthand for therapeutic futility. Used appropriately, it can support individualized decision-making, identify modifiable vulnerabilities, guide peri-procedural optimization, and align treatment intensity with patient priorities. Used poorly, it may reinforce ageism, disability bias, sex-based disparities, and inequitable access to invasive cardiovascular care. The central question is therefore not whether frail older adults should or should not undergo invasive management, but how frailty should be integrated into a proportional and patient-centered decision-making process.

This narrative review examines the role of frailty in invasive decision-making among older adults with ACS. We summarize the conceptual basis of frailty, review commonly used assessment tools, discuss its association with clinical and patient-centered outcomes, and analyze how frailty may inform the selection and intensity of invasive strategies. We also address the intersection between frailty, therapeutic bias, and healthcare inequities.

While previous reviews have primarily focused on frailty as a prognostic marker in older adults with acute coronary syndromes, the present review adopts a broader vulnerability-based perspective. Rather than examining frailty solely as a predictor of adverse outcomes, we explore how multidimensional vulnerability—including frailty, multimorbidity, cognitive function, functional status, social support, and patient goals—can inform invasive decision-making, guide proportional care, and support patient-centered management across the ACS continuum.

## 2. Search Strategy and Scope of the Review

This narrative review was designed to synthesize contemporary evidence and conceptual perspectives on frailty assessment and invasive decision-making in older adults with ACS. The review focuses on non-ST-segment elevation acute coronary syndromes and ST-segment elevation myocardial infarction where relevant, with particular attention to older patients, frailty, invasive coronary angiography, revascularization, clinical outcomes, patient-centered endpoints, and healthcare inequities.

A narrative rather than systematic approach was selected because the aim of this article is not to provide pooled estimates of treatment effect, but to integrate evidence across cardiology, geriatrics, interventional cardiology, and health services research. The review prioritizes studies addressing frailty assessment, outcomes after ACS, invasive versus conservative strategies in older adults, risk–treatment paradoxes, and the broader implications of vulnerability for therapeutic decision-making.

Relevant literature was identified through searches of PubMed, Embase, and Scopus up to June 2026 using combinations of the terms “acute coronary syndrome”, “frailty”, “older adults”, “geriatric assessment”, “coronary angiography”, “revascularization”, and “invasive strategy”. Randomized trials, observational studies, registry analyses, systematic reviews, meta-analyses, clinical guidelines, and position papers were considered. Priority was given to studies evaluating frailty assessment tools, invasive versus conservative management, and patient-centered outcomes in older adults with ACS.

The review is structured around three core questions: first, how should frailty be conceptualized and assessed in older adults with ACS; second, how does frailty influence clinical outcomes and the balance between benefit and harm of invasive management; and third, how can frailty be incorporated into a vulnerability-based model of cardiovascular care that supports equity, proportionality, and patient-centered decision-making.

## 3. Frailty as Biological Vulnerability in Cardiovascular Care

### 3.1. Conceptualizing Frailty Beyond Chronological Age

Frailty is increasingly recognized as a multidimensional state of heightened vulnerability resulting from age-related declines across multiple physiological systems, leading to reduced resilience to acute stressors [7]. In cardiovascular medicine, frailty has gained particular relevance because cardiovascular disease frequently acts as a physiological stress test capable of exposing limited biological reserve. ACS, in particular, may precipitate abrupt deterioration in functional status, cognition, mobility, nutritional state, and independence, especially in vulnerable older adults.

Importantly, frailty should not be considered synonymous with aging itself. Although frailty prevalence increases with age, chronological age alone provides limited information regarding physiological reserve, treatment tolerance, or recovery potential. Some octogenarians maintain preserved functional capacity and substantial resilience, whereas younger individuals with multimorbidity, sarcopenia, cognitive impairment, or disability may exhibit marked vulnerability. Frailty therefore reflects biological rather than chronological aging and captures dimensions of risk not adequately represented by conventional cardiovascular scores [2,7].

Frailty also differs conceptually from comorbidity and disability, although these conditions frequently coexist and interact. Comorbidity refers to the presence of multiple chronic diseases, whereas disability describes difficulty or dependency in performing activities of daily living. Frailty instead represents a state of decreased physiological reserve and impaired homeostatic capacity that predisposes individuals to disproportionate deterioration after relatively minor stressors [11]. This distinction is clinically important because frailty may be partially reversible, dynamic over time, and potentially modifiable through multidisciplinary interventions, optimization of nutrition, mobilization, medication review, and rehabilitation strategies.

Two principal conceptual models of frailty have dominated the literature. The frailty phenotype proposed by Fried and colleagues conceptualizes frailty as a biological syndrome characterized by weakness, slowness, exhaustion, low physical activity, and unintentional weight loss [12]. In contrast, the cumulative deficit model developed by Rockwood conceptualizes frailty as the progressive accumulation of deficits across multiple domains, including diseases, symptoms, disabilities, cognitive impairment, and psychosocial vulnerability [13]. Although these approaches differ methodologically, both identify patients at increased risk of adverse outcomes and reduced resilience.

In cardiovascular care, frailty has particular implications because it intersects with many processes directly relevant to ACS management. Frail patients are more likely to have sarcopenia, impaired mobility, chronic inflammation, renal dysfunction, malnutrition, polypharmacy, anemia, and cognitive impairment, all of which may influence procedural tolerance, antithrombotic safety, rehabilitation potential, and recovery trajectories [14,15]. Frailty may therefore affect not only prognosis but also the expected balance between benefit and harm associated with invasive treatment.

At the same time, frailty should not be interpreted as a static or deterministic condition. Functional trajectories among older adults are heterogeneous, and vulnerability may fluctuate over time according to acute illness, hospitalization, nutritional status, deconditioning, and social support. Some patients experience substantial recovery after ACS when timely revascularization, rehabilitation, and multidisciplinary care are provided. Others may deteriorate despite technically successful procedures due to limited physiological reserve or competing non-cardiovascular conditions. Recognizing this dynamic nature of frailty is essential to avoid equating vulnerability with therapeutic futility.

The increasing incorporation of frailty into cardiovascular care reflects a broader shift toward more individualized and patient-centered models of care. Traditional cardiovascular decision-making has largely focused on disease-specific variables such as coronary anatomy, ventricular function, biomarkers, and ischemic risk. Although these parameters remain indispensable, they do not fully capture the broader dimensions that determine recovery, tolerance to treatment, maintenance of independence, and quality of life in older adults. Frailty assessment therefore provides an opportunity to integrate biological reserve into therapeutic decision-making and to move beyond purely disease-centered models of ACS care.

### 3.2. Frailty and the Biological Response to Acute Coronary Syndromes

Acute coronary syndromes represent major physiological stressors that may overwhelm limited biological reserve in frail individuals. Beyond the ischemic insult itself, ACS frequently exposes underlying vulnerability through inflammatory activation, hemodynamic instability, immobilization, metabolic stress, polypharmacy, invasive procedures, and prolonged hospitalization. As a consequence, frail older adults are particularly susceptible to cascading complications that extend beyond the cardiovascular event alone.

Several biological mechanisms may explain the association between frailty and adverse outcomes in ACS. Frailty has been linked to chronic low-grade inflammation, endothelial dysfunction, impaired immune response, sarcopenia, neurohormonal dysregulation, and reduced cardiovascular reserve [16,17]. These alterations may impair the capacity to tolerate myocardial ischemia, recover after revascularization, respond to acute hemodynamic changes, or withstand procedural complications. Frail individuals may also exhibit diminished physiological adaptability, making them more vulnerable to delirium, acute kidney injury, bleeding, malnutrition, infection, and deconditioning during hospitalization.

Sarcopenia deserves particular attention in this context. Loss of skeletal muscle mass and function is highly prevalent among frail older adults and may contribute to reduced mobility, impaired rehabilitation potential, slower recovery, and increased procedural vulnerability [18]. Sarcopenia may also interact with nutritional deficiencies, inflammatory burden, and physical inactivity, creating a self-reinforcing cycle of progressive vulnerability after acute illness. In patients with ACS, prolonged bed rest and hospitalization may further accelerate functional decline, especially among those with limited baseline reserve.

Cognitive impairment is another important yet frequently underrecognized dimension of frailty in cardiovascular care. Cognitive dysfunction may affect symptom recognition, adherence to treatment, understanding of procedural risks and benefits, participation in shared decision-making, and post-discharge self-management [19]. Delirium is also common during ACS hospitalization in older adults and is associated with prolonged length of stay, loss of independence, institutionalization, and mortality [20]. Importantly, cognitive vulnerability may remain undetected if frailty assessment focuses exclusively on physical performance measures.

Frailty also interacts closely with multimorbidity and polypharmacy. Older adults with ACS frequently present with chronic kidney disease, anemia, diabetes mellitus, chronic obstructive pulmonary disease, heart failure, and prior cerebrovascular disease, conditions that may simultaneously increase ischemic risk, bleeding risk, procedural complexity, and competing non-cardiovascular mortality [21]. Invasive strategies in these patients therefore require a broader evaluation of anticipated benefit, treatment burden, recovery trajectory, and patient priorities rather than a purely anatomy-driven approach.

Importantly, frailty should not be interpreted solely as a predictor of poor outcomes. Frailty assessment may also identify domains that are potentially modifiable before or during hospitalization. Nutritional optimization, early mobilization, medication review, delirium prevention, rehabilitation, and multidisciplinary cardio-geriatric care may mitigate vulnerability and improve recovery trajectories [22]. This perspective is particularly relevant because invasive management decisions are often made rapidly during ACS admission, at a moment when frailty may be recognized but not yet adequately contextualized.

The interaction between frailty and ACS therefore extends beyond prognosis alone. Frailty influences the biological response to ischemia, tolerance to invasive treatment, capacity for recovery, and the meaning of clinical benefit itself. For some patients, survival without preservation of function or independence may not represent an acceptable outcome. Consequently, understanding frailty as a multidimensional and dynamic state of vulnerability is essential for achieving proportional and patient-centered cardiovascular care.

## 4. Frailty Assessment in Older Adults with Acute Coronary Syndrome

### 4.1. Why Frailty Assessment Matters in ACS

Frailty assessment has become increasingly relevant in the management of older adults with ACS because conventional cardiovascular risk models incompletely capture the multidimensional vulnerability that characterizes many elderly patients. Scores such as GRACE or TIMI provide valuable prognostic information regarding ischemic events and mortality, yet they do not adequately assess functional reserve, cognitive impairment, mobility limitation, nutritional status, or capacity for recovery after acute illness [23,24]. As a result, two patients with similar ischemic risk profiles may differ substantially in treatment tolerance, procedural resilience, and post-discharge trajectories.

Invasive management decisions in ACS frequently occur under conditions of clinical urgency and uncertainty. Clinicians must balance the potential ischemic benefit of coronary angiography and revascularization against the risks of bleeding, acute kidney injury, delirium, vascular complications, prolonged hospitalization, and functional decline. Frailty assessment may help contextualize these competing risks and support more individualized therapeutic decisions [25]. Frailty assessment should therefore be integrated in a manner that does not interfere with time-sensitive reperfusion strategies. In patients with STEMI or other emergent ACS presentations, guideline-recommended reperfusion should not be delayed to perform formal frailty assessment. Instead, vulnerability assessment should be used to optimize procedural planning, anticipate complications, guide multidisciplinary care, and support rehabilitation, discharge planning, and long-term recovery once the patient has been stabilized.

Importantly, frailty assessment should not be viewed as a mechanism for excluding patients from invasive care. Rather, it should function as a complementary clinical tool capable of refining risk stratification and identifying domains requiring optimization before, during, and after intervention. In some cases, frailty assessment may support a less invasive strategy because the anticipated procedural burden outweighs the likely benefit. In others, it may justify invasive management despite advanced age by demonstrating preserved biological reserve and favorable recovery potential.

Frailty assessment may also facilitate communication with patients and families. Discussions surrounding invasive management in older adults often involve uncertainty regarding prognosis, independence, quality of life, and acceptable trade-offs between survival and treatment burden. Objective assessment of vulnerability may support shared decision-making and help align therapeutic intensity with patient goals and expectations [26].

Despite growing recognition of its clinical importance, frailty assessment remains inconsistently implemented in routine cardiovascular care. Barriers include time constraints, lack of standardization, uncertainty regarding the optimal assessment tool, limited geriatric training among cardiologists, and concerns about feasibility during acute hospitalization [27]. Moreover, some clinicians may perceive frailty assessment as subjective or insufficiently actionable. These limitations partly explain why frailty remains underrecognized despite its strong association with adverse outcomes.

Another important challenge is the heterogeneity of frailty instruments themselves. Existing tools vary substantially in conceptual frameworks, complexity, required training, time burden, and domains assessed. The principal frailty instruments currently available for older adults with ACS, together with their feasibility and applicability in acute clinical settings, are summarized in Table 1. Some focus predominantly on physical performance, whereas others incorporate cognition, disability, comorbidity, nutrition, or psychosocial vulnerability. Consequently, different instruments may identify overlapping but not identical populations as frail [28]. This variability complicates comparisons across studies and contributes to uncertainty regarding which frailty tools are most appropriate for ACS care.

Nevertheless, the growing integration of frailty into cardiovascular research and guideline discussions reflects an important conceptual shift. Frailty assessment acknowledges that treatment decisions in older adults should not rely solely on disease-specific severity, but also on the broader context of biological reserve, functional resilience, and patient priorities. This transition from disease-centered to vulnerability-informed care may be particularly relevant in ACS, where invasive decisions often carry profound implications for both survival and subsequent quality of life. Reflecting this paradigm shift, the 2023 ESC Guidelines for Acute Coronary Syndromes [4] recommend routine assessment of frailty and comorbidity burden, suggesting tools such as the Clinical Frailty Scale and Charlson Comorbidity Index to support risk stratification. The guidelines emphasize that frailty should not be viewed as a contraindication to invasive management, but rather as one component of a holistic evaluation aimed at individualizing pharmacological and interventional treatment according to the balance between anticipated benefit, procedural risk, and patient-centered goals of care.

### 4.2. Frailty Assessment Tools in ACS

Multiple frailty instruments have been evaluated in older adults with cardiovascular disease, although no single tool has emerged as universally superior for ACS care [29]. Ideally, frailty assessment in acute cardiovascular settings should be rapid, reproducible, clinically meaningful, and feasible during hospitalization while still capturing the multidimensional nature of vulnerability.

One of the most widely used instruments in cardiovascular medicine is the Clinical Frailty Scale (CFS), originally developed within the Canadian Study of Health and Aging [30]. The CFS is a semiquantitative tool based on clinical judgment, functional capacity, dependence, mobility, cognition, and comorbidity, categorizing patients along a spectrum from very fit to terminally ill. Its simplicity and rapid bedside applicability have contributed to its increasing use in ACS and critical care populations. Several studies have demonstrated associations between higher CFS scores and mortality, procedural complications, delirium, prolonged hospitalization, and functional decline after ACS [8,31].

The frailty phenotype proposed by Fried and colleagues represents another influential model [12]. This approach defines frailty according to the presence of five physical criteria: weakness, slowness, exhaustion, low physical activity, and unintentional weight loss. Although extensively validated in geriatric populations, its implementation during acute hospitalization may be more challenging because some components require physical performance testing or baseline functional information that may not be readily available during ACS admission.

Frailty index models based on deficit accumulation offer a broader multidimensional perspective by quantifying the cumulative burden of deficits across clinical, functional, cognitive, and psychosocial domains [13]. These indices may provide highly granular characterization of vulnerability but are often less practical for rapid bedside assessment in acute cardiovascular settings.

Additional instruments have also been explored in ACS populations. The Edmonton Frail Scale incorporates cognition, social support, medication use, nutrition, mood, continence, and functional performance [32]. Gait speed has emerged as a simple marker of biological reserve and functional vulnerability associated with mortality and disability [33]. The Essential Frailty Toolset, initially developed in transcatheter aortic valve implantation populations, integrates lower extremity weakness, cognition, anemia, and hypoalbuminemia and may offer particular value because of its combination of objective biological and functional measures [34].

Comprehensive geriatric assessment (CGA) deserves special consideration because it extends beyond screening and provides multidimensional evaluation of medical conditions, medications, cognition, mobility, nutrition, psychosocial support, and functional capacity [35]. Although resource-intensive, CGA may be especially valuable in complex older adults with competing vulnerabilities, uncertainty regarding therapeutic goals, or discordance between chronological age and physiological reserve.

Importantly, frailty tools should not be interpreted in isolation. No instrument can fully capture the complexity of vulnerability, resilience, recovery potential, or patient priorities. Frailty assessment should therefore complement—not replace—clinical judgment. Overreliance on simplified scores risks reducing frailty to a binary label detached from the broader clinical context. This is particularly relevant because frailty is dynamic, multidimensional, and strongly influenced by acute illness, hospitalization, social support, and baseline functional trajectory. Among available frailty instruments, the Clinical Frailty Scale may be particularly attractive in ACS because it can be completed within seconds at the bedside and does not require physical performance testing, making it especially suitable for acute clinical settings [28].

The optimal approach to frailty assessment in ACS likely depends on the intended clinical purpose. Practical applications of frailty assessment across the ACS care pathway are summarized in Table 2. Rapid screening tools may facilitate bedside risk stratification and early identification of vulnerable patients, whereas multidimensional assessments may better inform rehabilitation planning, discharge decisions, and shared decision-making regarding invasive treatment intensity. Future efforts should therefore focus not only on identifying the “best” frailty scale, but also on integrating frailty assessment into practical and actionable models of cardiovascular care.

Among currently available frailty instruments, the Clinical Frailty Scale (CFS) is probably the most pragmatic tool for routine ACS practice. It can be completed rapidly at the bedside without physical performance testing, has been extensively validated in cardiovascular populations, and is feasible even in acutely ill patients. By contrast, although the Comprehensive Geriatric Assessment remains the reference standard for multidimensional evaluation, its implementation is more resource-intensive and should complement, rather than delay, acute cardiovascular management. Physical phenotype-based instruments such as the Fried Frailty Phenotype provide valuable prognostic information but are generally less practical during the acute phase of ACS because they require performance-based assessments that may not be feasible in unstable patients.

## 5. Frailty and Outcomes After Acute Coronary Syndromes

### 5.1. Evidence Supporting Invasive Management in Older Adults with Acute Coronary Syndromes

Before considering frailty as a prognostic or treatment-modifying factor, it is important to recognize that advanced age alone should not preclude an invasive strategy in patients with acute coronary syndromes. Older adults frequently have a high absolute ischemic risk and may derive clinically meaningful benefit from coronary angiography and revascularization when these procedures are otherwise indicated. Therapeutic decisions should therefore begin with the evidence supporting guideline-directed ACS management rather than with assumptions regarding limited benefit based on chronological age. Randomized evidence has demonstrated that invasive management can reduce recurrent ischemic events in selected older adults with non-ST-segment elevation acute coronary syndromes. In the After Eighty trial, an invasive strategy reduced the composite of myocardial infarction, urgent revascularization, stroke, and death compared with a conservative strategy in patients aged 80 years or older. More recently, the SENIOR-RITA trial enrolled patients aged 75 years or older, including individuals with frailty and multimorbidity. Although the invasive strategy did not significantly reduce the primary composite of cardiovascular death or nonfatal myocardial infarction, it was associated with fewer nonfatal myocardial infarctions, with a low incidence of procedural complications [36].

Contemporary meta-analyses of randomized trials similarly suggest that routine invasive management in older patients reduces recurrent myocardial infarction and the need for subsequent or urgent revascularization. However, a consistent reduction in all-cause or cardiovascular mortality has not been demonstrated [37]. These findings indicate that the principal benefits of an invasive strategy may lie in preventing recurrent ischemic events and future procedures rather than in universally improving survival. Accordingly, older age should not be used as a reason to withhold invasive evaluation, but neither should invasive management be applied indiscriminately without consideration of clinical presentation, comorbidity, frailty, procedural risk, and patient preferences.

An important limitation when interpreting this evidence is that medically managed patients with NSTE-ACS do not constitute a homogeneous clinical group. They include patients in whom coronary angiography is not performed, patients without obstructive coronary artery disease, and patients with significant coronary disease in whom revascularization is not pursued because of anatomical complexity, comorbidity, frailty, limited expected benefit, patient preferences, or clinical judgment. These distinct pathways are frequently combined under the label of “conservative management,” despite carrying different prognostic and therapeutic implications, as previously highlighted by Menozzi et al. [38] and the accompanying editorial [39].

Previous analyses have shown that medically managed patients are generally at higher baseline risk and experience worse outcomes than those undergoing revascularization. However, these associations cannot be interpreted as definitive evidence of a causal benefit from intervention because treatment allocation is strongly influenced by age, comorbidity, frailty, clinical instability, coronary anatomy, and perceived treatment futility. Consequently, observational comparisons are particularly vulnerable to selection bias and confounding by indication. Conservative management may represent appropriate proportional care in some patients, whereas in others it may reflect potentially avoidable undertreatment.

This heterogeneity also complicates the translation of randomized trial findings into routine practice. Many trials have excluded or underrepresented patients with severe frailty, cognitive impairment, extensive multimorbidity, limited life expectancy, or inability to provide consent. Furthermore, crossover to angiography for recurrent ischemia may reduce the contrast between randomized strategies. The available evidence should therefore support neither routine invasive treatment nor systematic therapeutic restriction based on age or frailty alone. Instead, it should inform individualized decisions grounded in clinical presentation, coronary anatomy, biological reserve, expected benefit, treatment burden, and patient preferences.

### 5.2. Frailty and Mortality After ACS

Frailty is consistently associated with increased mortality in older adults presenting with ACS, independently of chronological age and conventional cardiovascular risk scores [8,40]. Multiple observational studies and registry analyses have demonstrated that frail patients experience substantially higher rates of in-hospital mortality, short-term complications, and long-term adverse outcomes following both ST-segment elevation myocardial infarction and non-ST-segment elevation ACS [41].

Importantly, this association appears to persist even after adjustment for comorbidity burden, left ventricular dysfunction, renal impairment, and ischemic severity [31]. Frailty therefore captures dimensions of vulnerability that extend beyond traditional disease-centered prognostic markers. Reduced physiological reserve, impaired adaptability to acute stress, sarcopenia, chronic inflammation, and diminished recovery capacity likely contribute to the increased mortality observed among frail individuals after ACS.

Frailty may also influence outcomes indirectly through treatment patterns. Frail patients are less likely to undergo coronary angiography, revascularization, or aggressive secondary prevention strategies, even after accounting for age and comorbidities [42]. Consequently, part of the excess mortality associated with frailty may reflect undertreatment and therapeutic conservatism rather than biological vulnerability alone. This distinction is clinically relevant because it suggests that frailty should not automatically be interpreted as evidence of futility.

At the same time, frailty may modify the balance between procedural benefit and treatment-related harm. Frail individuals are more vulnerable to bleeding, vascular complications, contrast-induced kidney injury, prolonged immobilization, delirium, and deconditioning during hospitalization [43]. Mortality risk in these patients therefore emerges not only from the ischemic event itself but also from the cumulative physiological burden imposed by invasive procedures, hospitalization, and competing non-cardiovascular conditions.

Several studies have also suggested a graded relationship between frailty severity and adverse outcomes, with progressively worse survival across increasing frailty categories [44]. This supports the notion that frailty should not be conceptualized as a binary condition but rather as a continuum of biological vulnerability. Such a perspective may be particularly useful when discussing proportionality of care and expected recovery trajectories.

Notably, mortality may not fully capture the clinical significance of frailty in older adults with ACS. Some patients may survive hospitalization but experience substantial functional deterioration, cognitive decline, institutionalization, or loss of independence. Consequently, interpreting mortality outcomes in isolation risks underestimating the broader consequences of ACS in vulnerable older populations.

### 5.3. Frailty and Non-Fatal Adverse Outcomes

Beyond mortality, frailty is strongly associated with a wide range of adverse clinical and functional outcomes after ACS. Frail older adults are more likely to experience bleeding complications, acute kidney injury, delirium, falls, prolonged hospitalization, rehospitalization, disability, and institutionalization [8,45,46]. These events may profoundly affect quality of life, autonomy, and long-term recovery.

Bleeding risk is particularly relevant in contemporary ACS management because older frail patients frequently receive potent antithrombotic therapies while simultaneously exhibiting reduced physiological reserve, renal dysfunction, anemia, and polypharmacy [47]. Frailty has been associated with both major bleeding and clinically relevant non-major bleeding, complications that may trigger discontinuation of guideline-directed therapy, prolonged hospitalization, and downstream cardiovascular events.

Acute kidney injury represents another common complication in vulnerable patients undergoing invasive management. Frailty is associated with impaired renal reserve, chronic inflammation, sarcopenia, and increased susceptibility to hemodynamic instability, all of which may amplify the nephrotoxic effects of contrast exposure and acute illness [48]. Importantly, acute kidney injury after ACS is itself associated with increased mortality, prolonged hospitalization, and functional decline.

Delirium constitutes a particularly important but often underrecognized complication in older adults hospitalized with ACS. Frailty, cognitive impairment, sleep disruption, immobilization, polypharmacy, and acute physiological stress collectively increase vulnerability to delirium during hospitalization [49]. Delirium has major implications because it is associated not only with short-term morbidity but also with persistent cognitive decline, institutionalization, and reduced survival after discharge.

Functional deterioration is another central dimension of vulnerability after ACS. Even when cardiovascular stabilization is achieved, frail patients may experience marked declines in mobility, self-care capacity, and independence following hospitalization [50]. Bed rest, deconditioning, nutritional impairment, and prolonged recovery may accelerate disability trajectories that persist long after the acute event. In some patients, these functional consequences may be more clinically meaningful than recurrent ischemic events themselves.

Readmissions are also substantially more frequent among frail individuals after ACS [51]. These rehospitalizations often reflect the interaction between cardiovascular disease, multimorbidity, functional vulnerability, medication burden, and inadequate social support. Recurrent admissions may contribute to progressive decline, caregiver burden, and healthcare fragmentation.

Importantly, the association between frailty and adverse outcomes should not lead to deterministic assumptions regarding prognosis. Frailty increases vulnerability but does not uniformly predict poor recovery or lack of benefit from treatment. Some frail patients experience substantial improvement following timely revascularization, rehabilitation, optimization of medical therapy, and multidisciplinary care. The challenge therefore lies not in identifying frailty alone, but in understanding how vulnerability modifies expected benefit, recovery potential, and patient priorities within individualized clinical decision-making.

## 6. Frailty and Invasive Decision-Making: From Risk Stratification to Proportional Care

### 6.1. The Risk–Treatment Paradox in Older Adults with Acute Coronary Syndromes

Older adults presenting with ACS often represent a therapeutic paradox. They carry the highest absolute risk of recurrent ischemic events, heart failure, disability, and death, and therefore may derive substantial absolute benefit from effective evidence-based therapies. However, they are also less likely to undergo invasive evaluation and revascularization than younger patients, even after adjustment for clinical risk and comorbidity burden [52,53].

This phenomenon, commonly referred to as the risk–treatment paradox, has been consistently observed across ACS registries and healthcare systems. Patients at highest risk are frequently those least likely to receive guideline-recommended therapies. Advanced age, frailty, multimorbidity, cognitive impairment, and perceived procedural risk often influence physician decision-making, sometimes leading to therapeutic conservatism that exceeds what available evidence would support [54].

Historically, the underrepresentation of older adults in randomized clinical trials has contributed to uncertainty regarding the balance between benefit and harm of invasive management. Consequently, many treatment decisions in frail older adults have relied on extrapolation from younger and healthier populations. This evidence gap has encouraged both overtreatment and undertreatment, reflecting the difficulty of translating population-based evidence into individualized clinical decisions.

**Figure 1:**
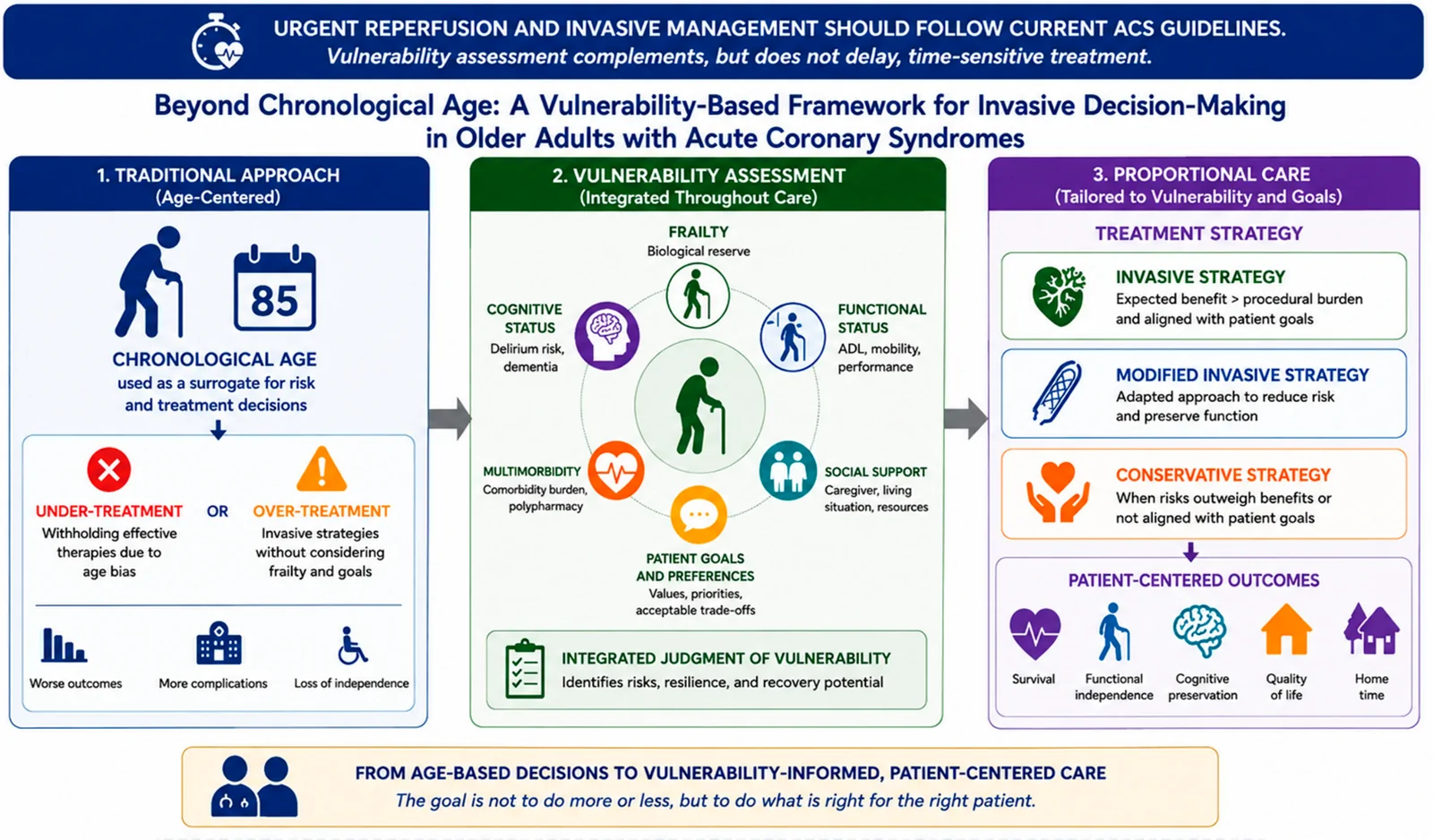
From Chronological Age to Vulnerability-Informed Decision-Making in Older Adults with Acute Coronary Syndromes. Traditional age-based approaches frequently use chronological age as a surrogate for risk and may contribute to both undertreatment and overtreatment. A vulnerability-based framework incorporates frailty, functional status, cognition, comorbidity burden, social support, and patient goals to guide proportional and individualized treatment strategies throughout the continuum of ACS care. Importantly, this conceptual framework is intended to complement, rather than delay, guideline-directed emergency management. In patients with time-sensitive presentations, particularly ST-segment elevation myocardial infarction (STEMI), multidimensional vulnerability assessment should not postpone urgent reperfusion or other evidence-based invasive therapies.

Importantly, chronological age alone has repeatedly demonstrated poor discrimination for treatment benefit. Studies evaluating invasive management in older adults have shown that selected elderly patients may derive meaningful reductions in recurrent ischemic events and improved outcomes despite advanced age [5,55]. These findings challenge the assumption that aging itself should constitute a barrier to invasive treatment. The transition from age-based decision-making toward vulnerability-informed proportional care is illustrated in Figure 1.

Frailty introduces additional complexity into this equation. Frail patients often exhibit greater procedural vulnerability, increased bleeding risk, higher rates of acute kidney injury, and lower physiological reserve. However, frailty also identifies individuals at particularly high baseline risk, raising the possibility that some may derive substantial benefit from successful treatment if procedural burden remains acceptable. The clinical challenge therefore lies not in deciding whether frailty is present, but in determining how frailty modifies the anticipated balance between benefit and harm.

Recent evidence has highlighted the limitations of adopting simplistic invasive-versus-conservative paradigms in frail populations. The MOSCA-FRAIL trial, which evaluated invasive versus conservative strategies in frail older adults with non-ST-segment elevation myocardial infarction, did not demonstrate a clear survival advantage associated with routine invasive management and underscored the importance of individualized decision-making [20]. Importantly, the study suggested that a routine invasive approach may not necessarily translate into better patient-centered outcomes, including time spent alive outside healthcare institutions. These findings reinforce the concept that greater treatment intensity is not invariably associated with greater clinical benefit in vulnerable older adults. Rather than supporting systematic treatment escalation or restriction, MOSCA-FRAIL highlights the need to individualize care according to biological reserve, competing risks, expected recovery, and patient priorities. Key studies evaluating invasive versus conservative strategies in older adults with ACS are summarized in Table 3.

Consequently, contemporary decision-making in older adults with ACS should move beyond age-based algorithms. The relevant question is no longer whether a patient is old or frail, but whether the expected clinical benefit of an invasive strategy justifies the procedural burden, aligns with patient priorities, and remains compatible with realistic recovery trajectories.

### 6.2. Frailty Should Inform, Not Dictate, Clinical Decisions

The growing recognition of frailty in cardiovascular medicine represents an important advance toward more individualized care. However, an unintended consequence of this progress is the risk of transforming frailty from a clinical assessment tool into a de facto mechanism of treatment restriction. Frailty identifies vulnerability, but vulnerability should not be equated with futility. This distinction is particularly important in ACS, where treatment decisions are often made rapidly and under conditions of uncertainty.

Historically, chronological age frequently influenced access to invasive cardiovascular therapies. Frailty assessment emerged partly in response to the limitations of age-based decision-making by providing a more nuanced understanding of biological reserve and expected recovery potential. Nevertheless, frailty itself may become vulnerable to misuse if interpreted as an automatic justification for withholding invasive treatment. Such an approach risks replacing one form of oversimplification with another.

Several observational studies have demonstrated that frail patients are less likely to undergo coronary angiography, percutaneous coronary intervention, and other evidence-based therapies, even after adjustment for clinical risk factors [1,42]. Although some of these differences may reflect appropriate individualized decision-making, others may represent therapeutic inertia, implicit bias, or excessive concern regarding procedural complications. Distinguishing between appropriate treatment limitation and potentially avoidable undertreatment remains one of the central challenges in contemporary geriatric cardiology.

Importantly, frailty should not be interpreted as a binary determinant of treatment eligibility. Patients with similar frailty scores may differ substantially in cognition, mobility, comorbidity burden, social support, nutritional status, symptom burden, and personal goals. Consequently, frailty should be interpreted within a broader multidimensional vulnerability assessment that integrates biological reserve, functional status, cognition, social circumstances, and patient preferences. This broader perspective allows clinicians to balance expected benefit, procedural burden, and recovery potential rather than relying on frailty alone to guide therapeutic decisions.

This distinction is particularly relevant because treatment benefit and procedural risk do not necessarily move in parallel. Patients with greater vulnerability often have both more to gain and more to lose from invasive management. While high ischemic risk may increase the potential benefit of revascularization, reduced physiological reserve may simultaneously increase the risk of bleeding, acute kidney injury, delirium, functional decline, and other complications. Clinical decisions therefore require balancing competing probabilities rather than applying rigid treatment thresholds.

Frailty may also intersect with broader healthcare inequities. Women, individuals with disability, socially vulnerable populations, and very old adults frequently experience lower rates of invasive cardiovascular treatment. When interpreted without appropriate clinical context, frailty may inadvertently reinforce existing disparities by providing an apparently objective justification for unequal treatment intensity [22]. Careful consideration of potential bias is therefore essential when incorporating frailty into therapeutic decision-making.

Ultimately, the purpose of frailty assessment is not to determine whether treatment should be offered, but to improve the quality of decision-making. Rather than functioning as a gatekeeping mechanism, frailty should facilitate discussions regarding anticipated benefits, potential harms, realistic recovery trajectories, and patient priorities. Used in this way, it supports proportional, individualized, and patient-centered cardiovascular care.

### 6.3. Balancing Benefit, Burden, and Recovery Potential

Traditional cardiovascular decision-making has largely focused on the probability of preventing adverse clinical events such as recurrent myocardial infarction, heart failure, or death. Although these outcomes remain critically important, they may not fully capture the dimensions of health that matter most to many older adults facing invasive management for ACS.

In younger populations, therapeutic success is frequently measured by survival or reduction in cardiovascular events. In frail older adults, however, the consequences of treatment often extend beyond these traditional endpoints. Functional independence, cognitive preservation, symptom burden, mobility, quality of life, and the ability to return to a meaningful living situation may be equally important—or even more important—than survival alone [3,56].

Consequently, invasive decision-making in older adults requires a broader framework that balances three interrelated dimensions: anticipated benefit, procedural burden, and recovery potential.

Anticipated benefit refers to the expected reduction in ischemic events, recurrent hospitalization, cardiovascular complications, or mortality associated with invasive treatment. Procedural burden encompasses the immediate and downstream consequences of intervention, including bleeding, acute kidney injury, delirium, prolonged hospitalization, functional decline, rehabilitation requirements, and caregiver burden. Recovery potential may also be assessed through patient-centered outcomes such as home time, defined as the number of days spent alive and outside healthcare institutions. In vulnerable older adults, home time may better reflect meaningful recovery than traditional cardiovascular endpoints alone.

Frailty influences all three domains simultaneously. A frail patient may have a particularly high ischemic risk and therefore substantial potential benefit from revascularization, while also facing increased procedural risk and reduced physiological reserve. Conversely, some vulnerable individuals may have limited life expectancy from competing non-cardiovascular conditions, reducing the probability that invasive treatment will translate into meaningful long-term benefit. The challenge is therefore not simply estimating procedural risk, but evaluating whether treatment is likely to produce outcomes that are meaningful to the individual patient.

This perspective highlights an important limitation of conventional risk models. Most cardiovascular scores estimate the probability of adverse events but provide limited information regarding post-treatment functional trajectories. Yet for many older adults, the distinction between survival with preserved independence and survival with severe disability may be central to treatment preferences. Prognostic discussions should therefore incorporate expected recovery patterns alongside traditional cardiovascular outcomes.

The concept of proportional care may be particularly useful in this context. Proportional care does not imply therapeutic limitations; rather, it seeks alignment between treatment intensity, biological reserve, expected benefit, and patient goals. Under this framework, invasive treatment may be entirely appropriate in selected frail patients when meaningful recovery remains achievable, while less intensive strategies may be reasonable when treatment burden is likely to outweigh anticipated benefit.

Ultimately, the goal of invasive decision-making in older adults with ACS should not be simply to prolong life, but to maximize the probability of outcomes that patients themselves consider worthwhile. Frailty assessment contributes to this process by helping clinicians understand not only the risk of dying, but also the likelihood of recovering.

### 6.4. Shared Decision-Making in Frail Older Adults

Shared decision-making is a cornerstone of contemporary cardiovascular care and assumes particular importance in older adults with ACS. In many clinical situations, the optimal therapeutic strategy cannot be determined solely by clinical variables, procedural risk estimates, or guideline recommendations. Instead, decisions frequently involve uncertainty regarding prognosis, competing risks, quality of life, recovery trajectories, and the relative value of different outcomes from the patient’s perspective.

This complexity becomes especially evident in frail individuals. While clinicians often focus on survival, recurrent ischemic events, or procedural success, older adults may prioritize other goals, including preservation of independence, maintenance of cognitive function, avoidance of institutionalization, symptom control, or minimizing treatment burden [56,57]. Consequently, therapeutic decisions that appear appropriate from a disease-centered perspective may not necessarily align with patient values and preferences.

Frailty assessment may facilitate these discussions by providing a more comprehensive understanding of vulnerability and expected recovery potential. Rather than functioning solely as a prognostic tool, frailty can help frame conversations regarding the likely benefits, burdens, and uncertainties associated with invasive treatment. This may be particularly valuable when evidence is limited, competing risks are substantial, or multiple reasonable management strategies exist.

Importantly, shared decision-making should not be viewed as a single conversation occurring immediately before a procedure. Older adults hospitalized with ACS often experience evolving clinical trajectories, changing priorities, and fluctuating functional status. Decisions regarding coronary angiography, revascularization, antithrombotic therapy, rehabilitation, and long-term care planning may therefore require ongoing reassessment throughout the course of treatment.

Cognitive impairment, sensory deficits, acute illness, and emotional distress may create additional challenges. Family members and caregivers frequently play a critical role in decision-making, particularly when patients have limited decisional capacity or require support in understanding complex medical information [58]. Nevertheless, clinicians should make every effort to preserve patient autonomy and ensure that individual values remain central to the decision-making process.

Shared decision-making is also closely linked to the concept of proportional care. The objective is not simply to identify the treatment associated with the lowest mortality risk, but rather to select the strategy most consistent with the patient’s goals, priorities, and acceptable trade-offs. For some individuals, maximizing longevity may represent the primary objective. For others, maintaining independence, avoiding prolonged hospitalization, or minimizing procedural burden may be equally or more important.

Ultimately, integrating frailty assessment into shared decision-making may help bridge the gap between evidence-based medicine and person-centered care. In this framework, frailty becomes a tool for understanding the individual patient rather than a label that determines treatment eligibility. Such an approach may be particularly valuable in ACS, where decisions frequently involve balancing uncertainty, competing risks, and diverse patient priorities.

### 6.5. Multidisciplinary Approaches and the Emerging Role of Cardio-Geriatrics

The management of frail older adults with ACS increasingly extends beyond the scope of traditional disease-centered cardiology. Although invasive decision-making has historically focused on anatomical findings, ischemic risk, and procedural feasibility, optimal care in older adults frequently requires consideration of functional status, cognitive reserve, social circumstances, and recovery potential. Consequently, multidisciplinary approaches are becoming increasingly important in the evaluation and management of vulnerable patients [34,42].

Frailty assessment illustrates this need particularly well. While cardiologists are uniquely positioned to evaluate cardiovascular risk and treatment options, many domains that influence outcomes in older adults fall outside conventional cardiovascular assessment. Functional impairment, cognitive vulnerability, nutritional status, polypharmacy, caregiver availability, and social support may substantially affect treatment tolerance and recovery following ACS. Comprehensive evaluation of these factors often requires collaboration among multiple healthcare professionals.

Geriatricians may contribute expertise in comprehensive geriatric assessment, identification of geriatric syndromes, functional evaluation, and prognostic assessment beyond cardiovascular disease alone. Nursing teams play a critical role in the detection of delirium, mobility limitations, medication-related complications, and evolving care needs during hospitalization. Rehabilitation specialists may help identify opportunities to preserve or restore functional independence, while pharmacists may assist in optimizing increasingly complex antithrombotic and cardiovascular treatment regimens. Social workers and case managers can address barriers related to caregiver support, healthcare access, and discharge planning [6,42].

The emergence of dedicated cardio-geriatric models reflects growing recognition that cardiovascular outcomes in older adults are influenced by factors extending beyond the acute cardiac event itself. In practical terms, multidisciplinary cardio-geriatric collaboration may function as a “Heart Team” adapted to the needs of older adults. Such models bring together cardiologists, geriatricians, nurses, rehabilitation specialists, pharmacists, patients, and caregivers to evaluate not only procedural feasibility but also functional reserve, cognitive vulnerability, social support, and patient-defined goals of care. This collaborative approach may be particularly valuable when the appropriateness of invasive management remains uncertain or when competing risks complicate therapeutic decision-making. Although organizational structures vary across healthcare systems, integrated collaboration between cardiology and geriatrics has been associated with improvements in care coordination, medication optimization, and patient-centered decision-making. Such approaches may be particularly valuable in patients presenting with frailty, multimorbidity, cognitive impairment, or uncertainty regarding the appropriateness of invasive treatment.

Importantly, multidisciplinary assessment should not be viewed as a mechanism for restricting access to cardiovascular therapies. Rather, its purpose is to improve the precision of clinical decision-making by identifying both vulnerabilities and sources of resilience that may influence treatment outcomes. In this context, frailty assessment becomes part of a broader effort to align therapeutic intensity with biological reserve, recovery potential, and patient priorities [1,41].

As populations continue to age, the integration of cardiology and geriatrics may become an increasingly important component of contemporary ACS care. Future healthcare models will likely require greater emphasis on collaborative decision-making, multidimensional assessment, and coordinated management strategies capable of addressing the complex needs of older adults with cardiovascular disease.

## 7. Frailty, Bias, and Healthcare Inequities

Frailty assessment has the potential to improve individualized cardiovascular care by providing a more nuanced understanding of biological reserve and vulnerability. However, like any clinical construct, frailty does not exist in isolation from the healthcare systems in which it is applied. The interpretation of frailty may be influenced by cognitive biases, cultural assumptions, organizational constraints, and broader social inequities, all of which can affect therapeutic decision-making.

One important concern is the potential overlap between frailty and ageism. Older adults have historically been underrepresented in cardiovascular clinical trials and are less likely to receive guideline-recommended therapies than younger patients, despite frequently having higher absolute cardiovascular risk [22]. Although frailty assessment was introduced partly to move beyond chronological age, there is a risk that frailty itself may be used as a surrogate for age-based treatment restriction. When frailty is interpreted primarily as evidence of limited life expectancy or poor treatment tolerance, clinicians may inadvertently reinforce therapeutic conservatism rather than improve individualized decision-making.

This concern is particularly relevant because frailty and treatment benefit are not mutually exclusive. Vulnerable patients often have the highest baseline risk and therefore may also have the greatest potential for absolute benefit from effective interventions. Decisions based solely on perceived vulnerability may therefore contribute to undertreatment, creating a paradox in which those most likely to experience adverse outcomes receive less intensive care.

Sex-related disparities may further complicate this landscape. Older women with ACS are generally older at presentation, more likely to be frail, and more likely to experience disability, multimorbidity, and social vulnerability than men [59]. These characteristics may contribute to lower rates of invasive evaluation and revascularization. Importantly, some observed treatment differences may reflect appropriate clinical individualization, whereas others may arise from implicit assumptions regarding treatment tolerance, expected benefit, or quality of life. Distinguishing between justified therapeutic adaptation and inequitable treatment remains a persistent challenge.

Frailty may also intersect with broader social determinants of health. Socioeconomic disadvantage, social isolation, limited health literacy, inadequate caregiver support, and restricted access to healthcare resources can influence both the development of frailty and the outcomes of cardiovascular disease [60]. These factors are rarely captured by traditional frailty instruments despite their substantial impact on treatment adherence, rehabilitation, recovery, and long-term prognosis.

Another limitation of current frailty frameworks is their tendency to emphasize deficits rather than resilience. Most frailty tools quantify vulnerability but provide limited information regarding protective factors such as social support, motivation, adaptive capacity, or access to rehabilitation resources. As a result, two patients with similar frailty scores may have markedly different capacities for recovery following an acute cardiovascular event.

These considerations support a broader conceptualization of vulnerability that extends beyond frailty alone. Vulnerability may be viewed as the result of interactions between biological reserve, functional capacity, cognition, social circumstances, healthcare access, and environmental context. Such a framework acknowledges that treatment decisions are influenced not only by patient characteristics but also by systemic factors that shape opportunities for recovery and access to care.

Ultimately, frailty assessment should promote equity rather than inadvertently reinforce disparities. Clinicians should remain aware of the potential for frailty to become a heuristic that simplifies complex decisions or justifies therapeutic limitations without sufficient consideration of individual circumstances. A vulnerability-informed approach may help ensure that frailty contributes to more personalized and equitable care rather than becoming another source of healthcare inequality.

## 8. Beyond Mortality: Patient-Centered Outcomes

Mortality has traditionally served as the principal endpoint in cardiovascular research and remains a critical measure of treatment effectiveness in ACS. The contrast between traditional cardiovascular endpoints and outcomes that may be more meaningful to older adults is shown in Table 4. However, in older adults, survival alone may not adequately reflect treatment success. Many patients value outcomes that extend beyond longevity, including preservation of independence, maintenance of cognitive function, symptom relief, quality of life, and the ability to remain engaged in meaningful daily activities [61,62].

This distinction becomes particularly relevant in frail populations. Frailty is associated not only with increased mortality but also with a higher risk of disability, functional decline, institutionalization, cognitive impairment, and loss of autonomy following hospitalization [50]. Consequently, two patients who survive an ACS may experience profoundly different recovery trajectories, with markedly different implications for well-being and quality of life.

Functional status represents one of the most important yet frequently overlooked outcomes in older adults. Preservation of mobility, self-care capacity, and independent living often carry equal or greater importance than traditional cardiovascular endpoints. Hospitalization itself may contribute to functional deterioration through immobilization, deconditioning, malnutrition, sleep disruption, and delirium, particularly among vulnerable individuals [63]. As a result, successful management of ACS should be evaluated not only according to survival but also according to the patient’s ability to recover meaningful function.

Cognitive outcomes deserve similar attention. Older adults with frailty are particularly susceptible to delirium during acute hospitalization, and episodes of delirium have been associated with subsequent cognitive decline, increased dependency, and reduced quality of life [49]. These consequences may persist long after the acute cardiovascular event has resolved and may substantially influence patient perceptions of treatment success.

The concept of *home time* has emerged as a potentially valuable patient-centered outcome in cardiovascular medicine. Home time refers to the number of days spent alive and outside healthcare institutions and may better reflect real-world recovery than mortality alone [64]. For many older adults, returning home and maintaining independence may be more meaningful than modest differences in survival probability. Similarly, avoidance of recurrent hospitalization may represent an important therapeutic goal that is not fully captured by conventional cardiovascular endpoints.

Quality of life should also occupy a central place in therapeutic decision-making. Patients frequently weigh symptom burden, treatment complexity, rehabilitation requirements, caregiver dependence, and expected functional recovery when evaluating potential interventions. Yet these considerations remain underrepresented in many clinical trials involving older adults with ACS. Greater incorporation of patient-reported outcomes may help bridge this gap and provide a more comprehensive understanding of treatment effectiveness.

Importantly, patient-centered outcomes do not replace traditional clinical endpoints but complement them. Survival remains essential, but it should be interpreted within the broader context of recovery, independence, cognition, and quality of life. A treatment strategy that prolongs survival without preserving outcomes that patients value may not always represent the optimal therapeutic choice.

As the population presenting with ACS continues to age, future research should increasingly incorporate outcomes that reflect what matters most to older adults themselves. Such an approach aligns closely with the principles of personalized medicine, shared decision-making, and proportional care. Ultimately, the goal of cardiovascular treatment should not simply be to extend life, but to maximize the likelihood of a life that remains meaningful to the individual patient.

## 9. Toward Vulnerability-Based Cardiovascular Care

The growing integration of frailty into cardiovascular medicine reflects a broader recognition that chronological age alone is insufficient to guide clinical decision-making in older adults. However, frailty itself should not be considered the final destination of this evolution. Rather, it represents one component of a more comprehensive framework of vulnerability that may better capture the complexity of older patients presenting with ACS [65].

Current clinical practice often remains dominated by disease-centered models that prioritize cardiovascular pathology and procedural risk. Although these approaches have generated substantial advances in acute cardiac care, they may inadequately address the multidimensional factors that influence outcomes in older adults. Biological reserve, multimorbidity, cognitive function, functional independence, social support, psychological resilience, and patient preferences all contribute to treatment tolerance and recovery potential [22]. No single frailty instrument can fully capture this complexity.

A vulnerability-based approach acknowledges that outcomes following ACS emerge from the interaction between the acute cardiovascular event and the patient’s underlying capacity to respond to physiological stress. Vulnerability should therefore be understood as a multidimensional construct encompassing biological, functional, cognitive, and social domains [7]. Frailty constitutes a central component of vulnerability, but it does not encompass all factors that influence prognosis or treatment benefit. A broader framework of vulnerability relevant to invasive decision-making is outlined in Table 5.

Within this framework, invasive decision-making moves beyond the traditional question of whether a patient is sufficiently fit or sufficiently frail to undergo treatment. Instead, clinicians are encouraged to consider how different dimensions of vulnerability influence the anticipated balance between benefit, burden, and recovery. Such an approach recognizes that vulnerability is not synonymous with futility and that even highly vulnerable patients may derive meaningful benefit from appropriately selected interventions.

Importantly, vulnerability-based care aligns naturally with the concept of proportional care. Proportional care seeks to match therapeutic intensity to biological reserve, expected benefit, recovery potential, and patient goals rather than applying uniform treatment strategies across heterogeneous populations [56]. In this model, invasive management is neither systematically pursued nor systematically withheld. Instead, treatment intensity is individualized according to the likelihood of achieving outcomes that are meaningful to the patient [56].

**Figure 2:**
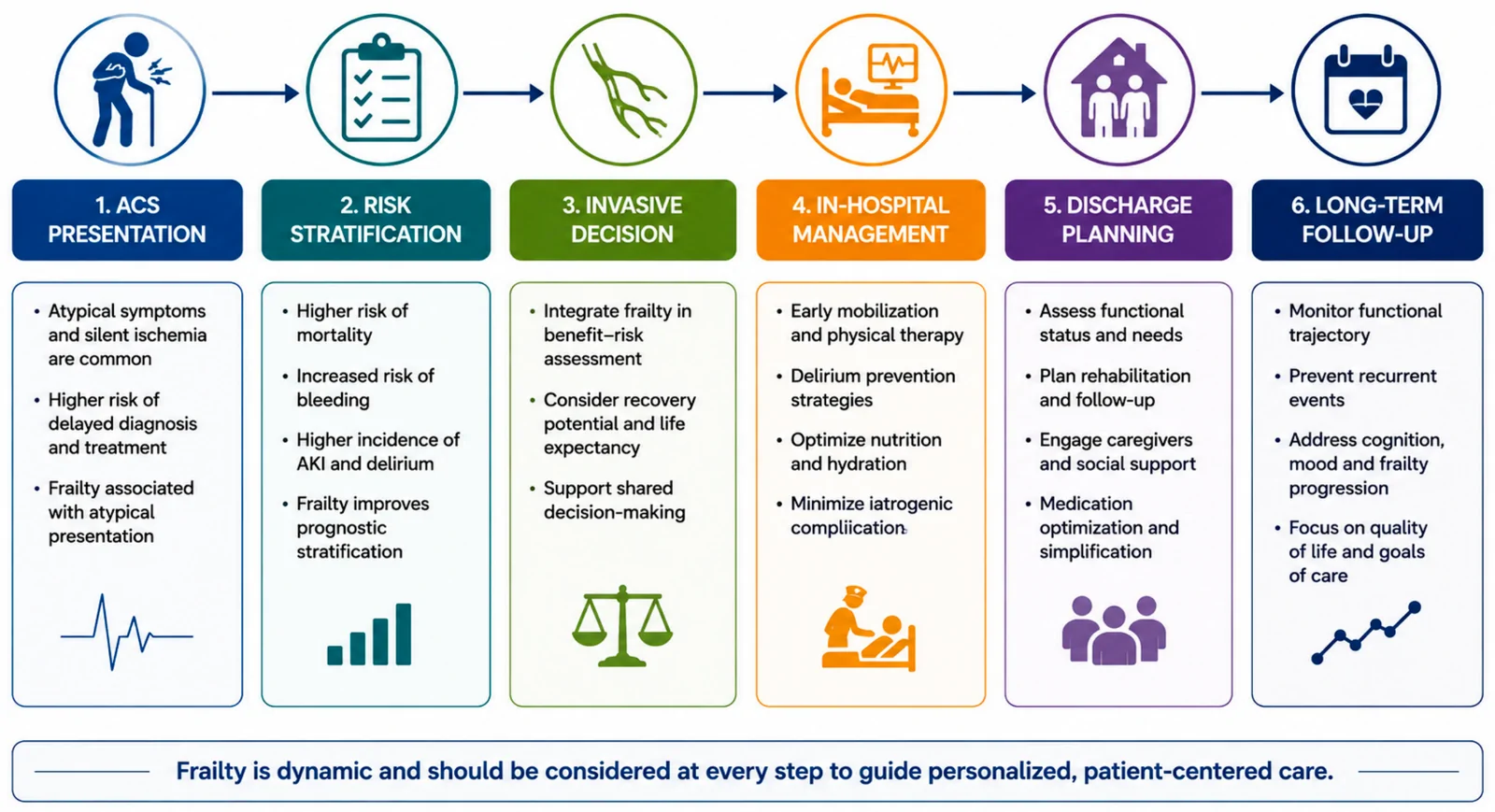
Frailty Across the Acute Coronary Syndrome Care Continuum. Frailty influences multiple stages of care, including clinical presentation, risk stratification, invasive decision-making, in-hospital management, discharge planning, and long-term follow-up. Assessment of vulnerability at each stage may facilitate patient-centered care and improve outcomes.

The implementation of vulnerability-based cardiovascular care will likely require greater collaboration between cardiology, geriatrics, rehabilitation specialists, nursing teams, patients, and caregivers [22]. Comprehensive assessment of vulnerability may facilitate more accurate prognostic evaluation, improve communication, support shared decision-making, and identify opportunities for targeted interventions aimed at enhancing resilience and recovery. A conceptual framework illustrating the transition from age-based cardiovascular care toward a vulnerability-informed approach is presented in Figure 2.

Future research should move beyond simply establishing that frailty predicts adverse outcomes. Greater emphasis should be placed on understanding how vulnerability modifies treatment benefit, identifying potentially reversible components of vulnerability, and developing clinical pathways that integrate multidimensional assessment into routine cardiovascular care. Studies should also incorporate patient-centered outcomes, including functional recovery, cognitive trajectories, quality of life, and maintenance of independence.

**Figure 3:**
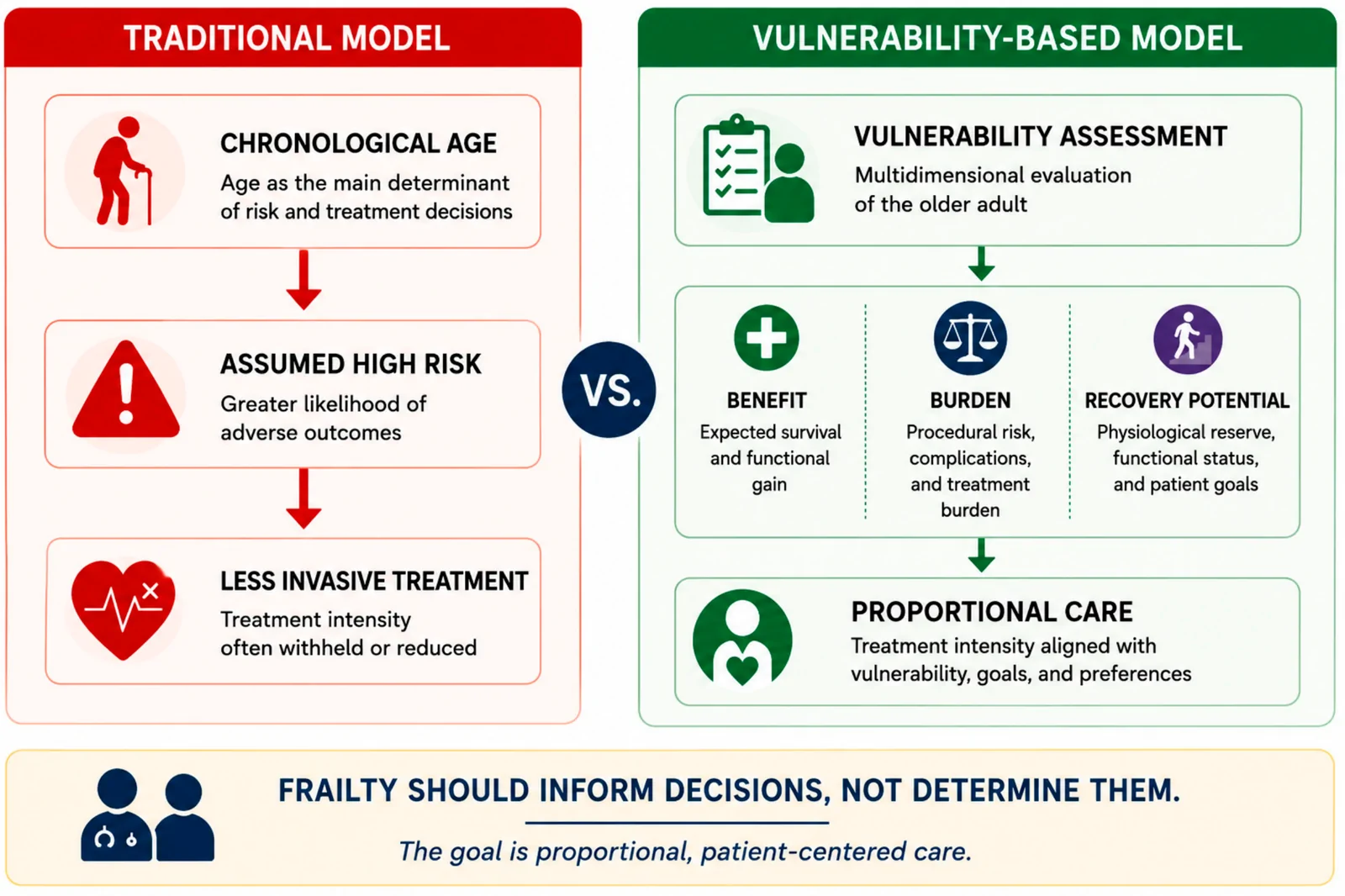
Traditional Versus Vulnerability-Based Models of Care in Older Adults with Acute Coronary Syndromes. In traditional models, chronological age frequently drives treatment decisions. In contrast, vulnerability-based care integrates expected benefit, treatment burden, recovery potential, and patient preferences to support proportional care aligned with individual goals.

Ultimately, the objective of cardiovascular care in older adults should not be merely to prolong survival, but to maximize the likelihood of meaningful recovery. Frailty assessment has provided an important step toward more individualized care. The next step may be the adoption of a broader vulnerability-based paradigm that better reflects the complexity, heterogeneity, and priorities of older adults with ACS, The influence of frailty across the continuum of ACS care is illustrated in Figure 3.

## 10. Future Directions

Despite the growing recognition of frailty in ACS, important knowledge gaps remain. Much of the existing literature has focused on the prognostic value of frailty assessment, whereas comparatively less attention has been devoted to understanding how frailty should influence therapeutic decision-making. Future research should move beyond risk prediction and explore how multidimensional vulnerability assessment can be integrated into clinical pathways that improve patient-centered outcomes.

Several priorities deserve particular attention. First, older adults with frailty remain underrepresented in cardiovascular clinical trials. Greater inclusion of vulnerable populations is essential to generate evidence that is directly applicable to the patients most commonly encountered in clinical practice. Future studies should evaluate not only survival but also functional recovery, cognitive trajectories, quality of life, and maintenance of independence.

Second, further work is needed to identify which dimensions of vulnerability are most relevant for decision-making in ACS. Current frailty instruments vary considerably in complexity and scope, and it remains unclear whether physical frailty alone adequately captures treatment tolerance and recovery potential. Future research should focus on developing and validating multidimensional vulnerability frameworks that integrate frailty, cognition, functional status, multimorbidity, social support, and patient goals. Such approaches may provide a more clinically meaningful assessment of biological reserve and recovery potential than frailty measures alone and may better support individualized treatment decisions in older adults with ACS.

Third, vulnerability should increasingly be evaluated as a dynamic rather than static construct. Acute illness, hospitalization, rehabilitation, and social circumstances may modify vulnerability over time. Longitudinal studies examining trajectories of frailty and recovery after ACS may provide valuable insights into resilience and reversibility.

Fourth, implementation research is needed to determine how frailty and vulnerability assessments can be incorporated into routine cardiovascular care without creating excessive complexity or delaying treatment. Pragmatic approaches that combine rapid screening with targeted multidimensional evaluation, ideally integrated within existing ACS pathways, may represent a feasible strategy. Future studies should also evaluate whether structured vulnerability assessment improves shared decision-making, treatment selection, and patient-centered outcomes in real-world clinical practice.

Finally, future investigations should examine how vulnerability-informed decision-making influences healthcare equity. Understanding the interaction between frailty, age, sex, disability, socioeconomic factors, and access to care may help ensure that vulnerability assessment promotes individualized treatment rather than inadvertently reinforcing existing disparities.

Ultimately, the next generation of research should focus not only on identifying vulnerable patients but also on developing interventions and decision frameworks capable of improving outcomes that matter most to older adults. The future challenge is no longer to determine whether frailty predicts adverse outcomes, but to understand how vulnerability modifies treatment benefit and how cardiovascular care can be adapted to maximize outcomes that matter most to older adults.

## 11. Conclusions

The increasing prevalence of ACS in older adults has challenged traditional approaches to cardiovascular decision-making. Chronological age alone provides an inadequate representation of the heterogeneity that characterizes this population, while frailty has emerged as a valuable construct for understanding biological reserve, vulnerability to stressors, and recovery potential.

Current evidence consistently demonstrates that frailty is associated with mortality, procedural complications, disability, cognitive decline, and loss of independence after ACS. However, frailty should not be interpreted as a binary determinant of treatment eligibility. Although vulnerability influences prognosis and treatment tolerance, it does not inevitably imply futility, nor should it serve as an automatic justification for restricting access to invasive therapies.

The greatest contribution of frailty assessment may lie not in predicting adverse outcomes, but in supporting more individualized clinical decisions. By informing the balance between anticipated benefit, procedural burden, and recovery potential, frailty can help clinicians move beyond age-based treatment paradigms and toward a more proportional approach to care. In this framework, treatment intensity is aligned with biological reserve, patient priorities, and realistic expectations regarding recovery rather than with chronological age alone.

At the same time, frailty represents only one dimension of vulnerability. Cognitive impairment, multimorbidity, functional dependence, social circumstances, and healthcare inequities also influence outcomes and therapeutic decision-making. A broader vulnerability-based framework may therefore provide a more comprehensive understanding of older adults with ACS and better reflect the complexity of real-world clinical practice.

Ultimately, the goal of cardiovascular care in older adults with ACS is not simply to prolong survival but to maximize meaningful recovery while respecting individual goals and preferences. Frailty should not be viewed as a contraindication to invasive treatment but as one component of a broader vulnerability-based framework that supports proportional, individualized, and patient-centered care. Future research should focus on integrating multidimensional vulnerability assessment into clinical pathways and evaluating its impact on patient-centered outcomes.

## Figures and Tables

**Table 1:** Common Frailty Assessment Tools in Older Adults with Acute Coronary Syndromes.

Tool	Domains Assessed	Time Required	Main Strengths	Main Limitations	Feasibility in Acute ACS	Recommended Clinical Use in ACS
Clinical Frailty Scale (CFS)	Functional status, dependence, cognition	<1 min	Rapid bedside assessment; extensively validated; highly feasible in acute settings	Subjective; potential inter-observer variability	Excellent	Preferred first-line screening tool for routine clinical practice
Fried Frailty Phenotype	Weight loss, exhaustion, weakness, slowness, low physical activity	5–10 min	Widely validated biological frailty model	Requires physical performance testing; difficult during acute illness	Limited	Best suited for stable patients and research settings
Frailty Index (Rockwood)	Accumulation of multidimensional deficits	Variable	Comprehensive characterization of biological aging	Time-consuming; limited bedside practicality	Limited	Comprehensive risk stratification when sufficient clinical data are available
Edmonton Frail Scale	Physical, cognitive, social, and functional domains	5–10 min	Multidimensional and relatively simple	Less extensively studied in ACS populations	Moderate	Alternative multidimensional screening tool when resources permit
Essential Frailty Toolset (EFT)	Physical performance, cognition, hemoglobin, albumin	3–5 min	Objective and clinically practical; integrates biological and functional domains	Validation mainly derived from structural heart disease populations	Good	Particularly useful in patients undergoing invasive cardiovascular procedures; promising for selected ACS patients
Comprehensive Geriatric Assessment (CGA)	Functional, cognitive, nutritional, social, and medical domains	>30 min	Reference standard for multidimensional assessment	Resource-intensive; difficult to implement routinely during acute ACS	Excellent after stabilization; limited in the acute phase	Reference standard after initial stabilization to guide individualized management, rehabilitation, discharge planning, and long-term care

Comparison of the principal frailty assessment instruments applicable to older adults with acute coronary syndromes. The Clinical Frailty Scale (CFS) is currently the most pragmatic instrument for routine bedside assessment in acute ACS because of its simplicity, rapid administration, and extensive cardiovascular validation. Comprehensive Geriatric Assessment (CGA) remains the reference standard for multidimensional evaluation but is generally performed after initial stabilization and should not delay guideline-directed acute management. Abbreviations: ACS, acute coronary syndrome; CFS, Clinical Frailty Scale; EFT, Essential Frailty Toolset; CGA, Comprehensive Geriatric Assessment.

**Table 2:** Practical Considerations for Frailty Assessment Across the ACS Care Pathway.

Clinical Stage	Potential Contribution of Frailty Assessment
ACS presentation	Identification of vulnerable patients at increased risk of atypical presentation, delayed diagnosis, and adverse outcomes
Risk stratification	Refinement of mortality, bleeding, delirium, acute kidney injury, and functional decline risk estimation
Invasive decision-making	Evaluation of expected benefit, procedural burden, treatment tolerance, and recovery potential
In-hospital management	Identification of patients requiring delirium prevention, nutritional support, medication review, and early mobilization
Discharge planning	Assessment of rehabilitation needs, caregiver support, social vulnerability, and discharge destination
Long-term follow-up	Monitoring frailty progression, functional recovery, cognitive outcomes, quality of life, and recurrent hospitalizations

**Table 3:** Critical Appraisal of Major Studies Evaluating Invasive and Conservative Management in Older Adults with NSTE-ACS.

Study	Population and Frailty Representation	Design	Compared Strategies	Principal Outcomes	Main Limitations	Clinical Implications
After Eighty	≥80 years with NSTEMI or unstable angina; selected trial population	Randomized, open-label trial	Routine invasive vs. conservative strategy	Invasive strategy reduced the composite ischemic endpoint, largely through fewer myocardial infarctions and urgent revascularizations	Limited representation of patients with severe frailty; benefit attenuated with increasing age; open-label design	Advanced age alone should not preclude invasive evaluation; benefit appears principally ischemic rather than clearly mortality-related
Italian Elderly ACS Trial	Older adults with NSTE-ACS; subgroup heterogeneity	Randomized trial	Early invasive vs. initially conservative strategy	No significant overall reduction in the primary composite; possible benefit in selected higher-risk subgroups	Limited sample size and statistical power; subgroup findings exploratory	Routine invasive management may not provide uniform benefit across all older adults
MOSCA	Patients ≥70 years with substantial comorbidity and NSTE-ACS	Randomized pilot trial	Invasive vs. conservative strategy	No clear overall survival advantage; possible early reduction in ischemic events	Small sample; limited power; high competing non-cardiovascular risk	Multimorbidity may attenuate long-term benefit and should be incorporated into treatment decisions
MOSCA-FRAIL	≥70 years, NSTEMI, CFS ≥ 4	Multicenter randomized trial	Routine invasive vs. conservative/watchful-observation strategy	No improvement in days alive and out of hospital or ischemic events at 1 year	Premature termination; only 167 patients; clinicians excluded patients for whom either strategy was considered clearly inappropriate	In frail patients, routine invasive management cannot be assumed to improve patient-centered outcomes; individualized selection remains essential
SENIOR-RITA	≥75 years with NSTEMI; mean age 82; 32% frail; multimorbidity permitted	Large multicenter randomized trial	Invasive strategy plus medical therapy vs. conservative medical therapy	No significant reduction in cardiovascular death or nonfatal MI; fewer nonfatal MIs; procedural complications < 1%	Open-label; conservative arm allowed angiography for recurrent ischemia; results may not apply to unstable STEMI or patients requiring mandatory intervention	Routine invasive management is safe in selected older adults but does not uniformly improve the primary clinical outcome; treatment should be individualized
SENIOR-NSTEMI	Older adults with NSTEMI in routine clinical practice	Observational cohort	Invasive vs. non-invasive management	Invasive management associated with lower adjusted mortality	Residual confounding, immortal-time bias and selection of fitter patients cannot be excluded	Supports potential benefit but cannot establish causality
LONGEVO-SCA	Very old patients with ACS; systematic geriatric assessment	Prospective observational registry	Treatment selected in clinical practice	Frailty independently predicted mortality and influenced treatment allocation	Observational design; limited sample size; heterogeneous ACS management	Demonstrates the prognostic value of frailty but not whether frailty modifies the causal effect of invasive treatment
Medically managed NSTE-ACS cohorts	Heterogeneous patients without angiography, without obstructive CAD, or with CAD not revascularized	Registries and observational studies	Medical management without routine revascularization	Higher event rates and less frequent use of guideline-directed pharmacotherapy	Strong confounding by indication; heterogeneous reasons for non-revascularization	“Conservative management” should not be treated as a single strategy; reasons for non-invasive care must be explicitly characterized

**Table 4:** Traditional Cardiovascular Outcomes versus Patient-Centered Outcomes in Older Adults with Acute Coronary Syndrome.

Traditional Outcomes	Patient-Centered Outcomes
Mortality	Functional independence
Recurrent myocardial infarction	Home time
Revascularization success	Cognitive preservation
Major adverse cardiovascular events	Quality of life
Length of stay	Return to previous living situation
Readmission	Symptom burden

**Table 5:** Components of Vulnerability Beyond Frailty Relevant to Invasive Decision-Making in Acute Coronary Syndromes.

Domain	Examples	Potential Impact on Decision-Making
Frailty	CFS, Fried phenotype, gait speed	Treatment tolerance, recovery potential
Comorbidity burden	CKD, COPD, heart failure, cancer	Competing risks and life expectancy
Cognitive function	Dementia, mild cognitive impairment, delirium risk	Shared decision-making and adherence
Functional status	ADLs, mobility, independence	Post-discharge recovery and rehabilitation
Social vulnerability	Living alone, caregiver support, health literacy	Recovery trajectory and care planning
Patient goals	Longevity, independence, symptom relief	Selection of proportional care strategy

## Data Availability

No new data were created or analyzed in this study. Data sharing is not applicable to this article.
